# Artificial intelligence applications in hypertrophic cardiomyopathy: turns and loopholes

**DOI:** 10.1093/ehjdh/ztaf086

**Published:** 2025-07-25

**Authors:** Giorgia Panichella, Manuel Garofalo, Laura Sasso, Alessandra Milazzo, Alessandra Fornaro, Josè Manuel Pioner, Alfonso Bueno-Orovio, Mark van Gils, Annariina Koivu, Luca Mainardi, Virginie Le Rolle, Felix Agakov, Maurizio Pieroni, Katriina Aalto-Setälä, Jari Hyttinen, Iacopo Olivotto, Annamaria Del Franco

**Affiliations:** Department of Experimental and Clinical Medicine, University of Florence, Viale Morgagni, 63, Florence 50134, Italy; Cardiomyopathy Unit, Careggi University Hospital, Largo Giovanni Alessandro Brambilla, 1, Florence 50134, Italy; Department of Experimental and Clinical Medicine, University of Florence, Viale Morgagni, 63, Florence 50134, Italy; Cardiomyopathy Unit, Careggi University Hospital, Largo Giovanni Alessandro Brambilla, 1, Florence 50134, Italy; Cardiomyopathy Unit, Careggi University Hospital, Largo Giovanni Alessandro Brambilla, 1, Florence 50134, Italy; Department of Experimental and Clinical Medicine, University of Florence, Viale Morgagni, 63, Florence 50134, Italy; Cardiomyopathy Unit, Careggi University Hospital, Largo Giovanni Alessandro Brambilla, 1, Florence 50134, Italy; Cardiomyopathy Unit, Careggi University Hospital, Largo Giovanni Alessandro Brambilla, 1, Florence 50134, Italy; Department of Biology, University of Florence, Florence, Italy; Department of Computer Science, University of Oxford, Oxford, United Kingdom; Faculty of Medicine and Health Technology, Tampere University, Tampere, Finland; Faculty of Medicine and Health Technology, Tampere University, Tampere, Finland; Department of Electronics, Information and Bioengineering, Politecnico di Milano, Milan, Italy; Department of Cardiology, University of Rennes, CHU Rennes, Inserm, LTSI—UMR 1099 Rennes F-35000, France; Pharmatics Limited, Edinburgh, United Kingdom; Department of Experimental and Clinical Medicine, University of Florence, Viale Morgagni, 63, Florence 50134, Italy; Cardiomyopathy Unit, Careggi University Hospital, Largo Giovanni Alessandro Brambilla, 1, Florence 50134, Italy; Faculty of Medicine and Health Technology, Tampere University, Tampere, Finland; Heart Hospital, Tampere University Hospital, Finland; Faculty of Medicine and Health Technology, Tampere University, Tampere, Finland; Heart Hospital, Tampere University Hospital, Finland; Department of Experimental and Clinical Medicine, University of Florence, Viale Morgagni, 63, Florence 50134, Italy; Cardiomyopathy Unit, Careggi University Hospital, Largo Giovanni Alessandro Brambilla, 1, Florence 50134, Italy; Cardiology Unit, Meyer University Hospital, Florence, Italy; Cardiomyopathy Unit, Careggi University Hospital, Largo Giovanni Alessandro Brambilla, 1, Florence 50134, Italy

**Keywords:** artificial intelligence, Hypertrophic cardiomyopathy, Machine learning, Left ventricular hypertrophy, Deep learning, Digital-twin

## Abstract

Hypertrophic cardiomyopathy (HCM) is a heterogeneous disease where, despite recent advances, accurate diagnosis, risk stratification, and personalized treatment remain challenging. Artificial intelligence (AI) offers a transformative approach to HCM by enabling rapid, precise analysis of complex data. This article reviews the current and potential applications of AI in HCM. AI enhances diagnostic accuracy by analysing electrocardiograms, echocardiography, and cardiac magnetic resonance images, differentiating HCM from other forms of left ventricular hypertrophy, identifying subtle phenotypic variations, and standardizing myocardial fibrosis assessment. Multimodal AI-driven approaches improve risk stratification, therapeutic decision-making, and monitoring of both established and novel therapies, such as cardiac myosin inhibitors. Emerging AI-driven *in silico* trials and digital twin platforms highlight the potential of combining data-driven and knowledge-based AI with biophysical models to simulate patient-specific disease trajectories, supporting preclinical evaluation and personalized care. As a multidisciplinary case study, the SMASH-HCM consortium is presented to illustrate how digital twin technologies and hybrid modelling can bring AI into clinical practice. Integration of genetic data further enhances AI's ability to identify at-risk individuals and predict disease progression. However, widespread AI applications raise concerns regarding data privacy, ethical considerations, and the risk of biases. Guidelines for researchers and developers—e.g. on trustworthy AI, regulatory frameworks, and transparent policies—are essential to address these possible pitfalls. As AI rapidly evolves, it has the potential to revolutionize drug discovery, disease management, and the patient journey in HCM, making interventions more precise, timely, and patient-centred.

## Graphical Abstract

**Graphical Abstract:**
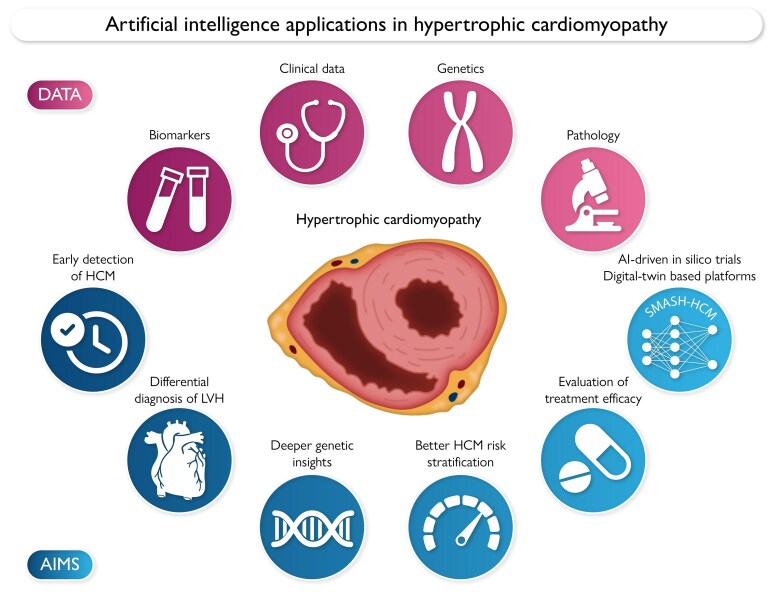

## Introduction

Hypertrophic cardiomyopathy (HCM) is a genetically determined heart disease characterized by unexplained left ventricular hypertrophy (LVH) that is not attributable to conditions such as hypertension or aortic stenosis.^1^ It is often associated with allelic variants in sarcomere proteins, leading to disorganized myocardial architecture, increased myocardial stiffness, and impaired ventricular relaxation.^1^ Given its phenotypic heterogeneity and variability in treatment response, the management of HCM requires a patient-tailored approach encompassing symptom control, risk stratification for sudden cardiac death (SCD), and definition of therapeutic strategies ranging from pharmacological management, septal reduction therapies, and implantable cardioverter-defibrillators (ICDs).^1^

In recent years, advances in artificial intelligence (AI), enabled by significant increases in computational power, the availability of large and diverse databases, and the development of new theories and algorithms, have added a new dimension to the diagnosis, risk stratification, and management of HCM. AI-based algorithms hold the potential to significantly improve diagnostic accuracy in HCM by evaluating multiple data, like electrocardiogram (ECG), echocardiogram, and cardiac magnetic resonance (CMR) imaging (*Graphical Abstract*).

We here review novel, AI-driven approaches aimed at improving the detection of subtle phenotypic variations, predicting clinical outcomes, and evaluating response to treatment. We conducted a non-systematic search of the PubMed database using the keywords ‘artificial intelligence AND hypertrophic cardiomyopathy’; studies were selected based on their clinical relevance, methodological quality, and impact on the field. The SMASH-HCM project is also introduced as a multidisciplinary implementation initiative, integrating AI research, clinical data, and biophysical modelling to create real-world digital health solutions in HCM.

## Overview of artificial intelligence

An AI system refers to a machine-based system that is designed to operate with varying levels of autonomy, that may exhibit adaptiveness, and that infers, from the input it receives, how to generate predictions, content, or recommendations.^2^ The various branches of AI have many applications in cardiology and are expected to change the landscape of disease diagnosis, treatment planning, and patient management.

Machine learning (ML) is a branch of AI that focuses on creating algorithms that can learn from data and make predictions or decisions without being explicitly programmed to perform those tasks.^3^ Deep learning (DL) is a specialized subset of ML that uses multi-layer artificial neural networks to model complex patterns in large datasets. These deep neural networks are particularly effective when trained on extensive, well-annotated datasets.^3^ Convolutional neural networks (CNNs) are a type of DL model specifically designed to process structured grid data, such as ECG and images. ML and DL, by combining large datasets of signals with other sources of patient information—such as genetic profiles, clinical history, biophysical computer models, and unstructured data—may enhance phenotyping, improve arrhythmic risk prediction, and uncover the complex mechanisms linking genetic backgrounds, structural abnormalities, and functional phenotypes.^4^

Natural language processing (NLP) is an AI discipline that focuses on the interaction between computers and human (natural) language.^3^ NLP techniques enable machines to understand, interpret, and generate human language, which is particularly useful for extracting information from unstructured text data in electronic health records.^3^

**Figure 1:**
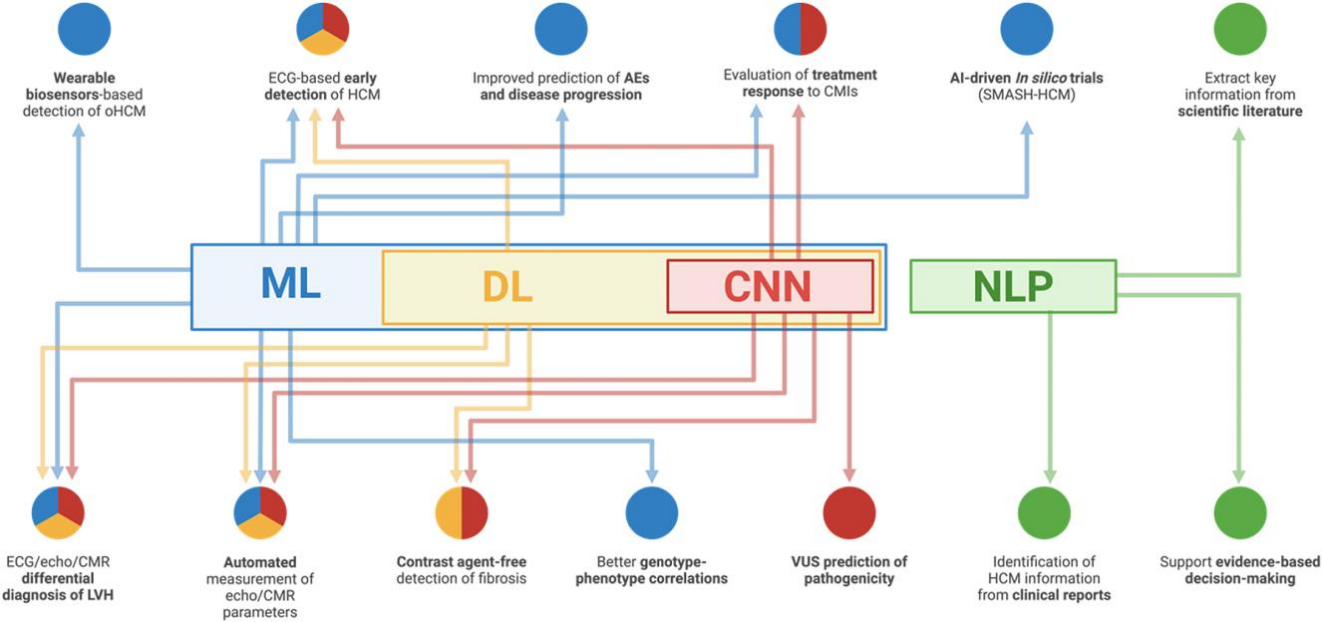
Diagram illustrating AI taxonomy and the different applications in HCM. The figure illustrates the hierarchy of AI models, with ML encompassing DL, which includes CNNs, and NLP as a separate approach. AI applications in HCM, such as risk stratification, diagnosis, imaging analysis, genotype prediction, and patient management, are linked to their respective models using colour-coded arrows: *blue for ML*, *yellow for DL*, *red for CNNs*, and *green for NLP*. Overlapping connections indicate that multiple models can contribute to the same application, highlighting AI's versatility in HCM management. CNN, convolutional neural network; DL, deep learning; ECG, electrocardiogram; AI, artificial intelligence; CMI, cardiac myosin inhibitor; LVH, left ventricular hypertrophy; CMR, cardiac magnetic resonance; HCM, hypertrophic cardiomyopathy; ML, machine learning; NLP, natural language processing; oHCM, obstructive hypertrophic cardiomyopathy.

**Figure 2:**
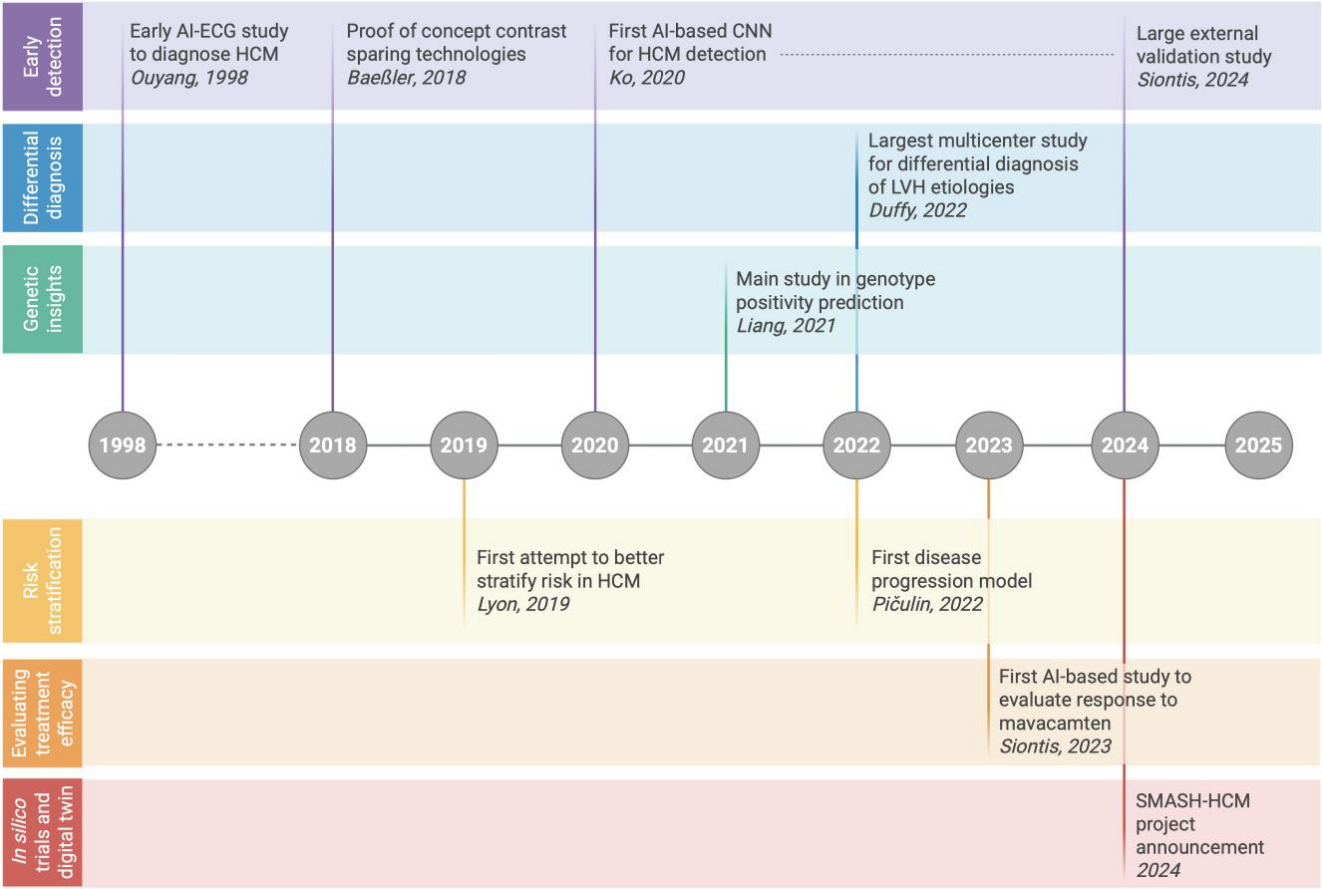
Timeline of key milestones in AI applications for HCM. This timeline illustrates selected landmark studies showcasing the evolution of artificial intelligence (AI) in the diagnosis, risk stratification, genetic profiling, and treatment evaluation of hypertrophic cardiomyopathy (HCM). Studies were included based on their innovation (e.g. first to introduce a novel AI application) or relevance (e.g. large-scale, multicentre, or externally validated work). The timeline is organized across thematic domains—early detection, differential diagnosis, genetic insights, risk stratification, treatment efficacy, and *in silico* trials/digital twin technologies—highlighting the progressive integration of AI into various aspects of HCM management. AI, artificial intelligence; CNN, convolutional neural network; ECG, electrocardiogram; LVH, left ventricular hypertrophy; HCM, hypertrophic cardiomyopathy.

By integrating AI into clinical workflows, healthcare providers can access a more concise and comprehensive synthesis of available data, facilitating a holistic understanding of HCM (*Figure 1*). This can lead to improved patient outcomes through personalized treatment approaches and targeted risk management. *Table 1* summarizes key studies on AI in HCM, whereas *Table 2* details their methodological features and performance metrics. To illustrate the evolution of AI applications in this field, we have also included a graphical timeline (*Figure 2*).

**Table 1:** Summary of main studies investigating the application of artificial intelligence to different diagnostic techniques in HCM

Data source	Early detection	Differential diagnosis	Deeper genetic insights	Better risk stratification	Evaluation of treatment efficacy
ECG and related techniques	Diagnosis of HCM at ECG^7–9,43^ or wearable biosensors^11^ ECG-based identification of hypertrophic segments^5^				Tracking of treatment response in HCM patients receiving mavacamten^43^
Echocardiography	HCM diagnosis among individuals with LVH^13^	Differentiation of common aetiologies of LVH (i.e. HHD, HCM, and CA)^20–23^	Prediction of positive genotype^27^		
CMR	Automated quantification of LV volumes and function^14^ Automated measurement of T1, ECV,^15^ and MWT^16^ Automated myocardial segmentation and quantification of myocardial fibrosis^17^ Contrast-sparing diagnosis of HCM^18,19^	Distinguishing different cardiomyopathies or causes of LVH^24,25^	Prediction of positive genotype^29^ Better genotype—phenotype correlations^30^	LGE-based arrhythmic risk stratification^41^	
Multimodal evaluation		Determination of LVH aetiology based on joint interpretation of 12-lead ECG and echocardiogram^26^	Prediction of positive genotype^28^ VUS pathogenicity prediction^32^	Improvement of existing HCM risk-stratification tools^34^ Development of new HCM risk-stratification tools in terms of AF,^35^ VAs,^38^ HF and disease progression,^36,39^ and MACE^33,37,40,42^	Multiparametric evaluation of mavacamten effects^44^ and therapeutic response^45^

CA, cardiac amyloidosis; CMR, cardiac magnetic resonance; CVD, cardiovascular disease; ECV, extracellular volume; HF, heart failure; HHD, hypertensive heart disease; HCM, hypertrophic cardiomyopathy; LGE, late gadolinium enhancement; LVH, left ventricular hypertrophy; MACE, major adverse cardiovascular events; MWT, maximum wall thickness; SCD, sudden cardiac death; VUS, variant of unknown significance.

**Table 2:** Summary of key studies on AI applications in HCM: methodological features and performance metrics

Study (1st author, year)	Sample size	N° centres	External validation	Input modalities	Aim	Quantitative measures	Reproducibility disclosed/Public data available	Clinical validation
*Ouyang, 1998^5^*	79 pts with HCM	Single-centre	No	ECG	Using ECGs to diagnose the hypertrophic portions of HCM	—	No	Retrospective
*Green, 2019^11^*	19 HCM pts vs. 64 healthy volunteers	Single-centre	No	PPG signals	oHCM detection	C-statistics 0.99 (95% CI 0.99–1.0)	No	Retrospective
*Tison, 2019^6^*	36 186 ECGs from general population	Single-centre	No	ECG	ECG-based detection of HCM	AUC 0.91 (95% CI 0.90–0.92)	No	Retrospective
*Ko, 2020^7^*	2448 HCM pts vs. 51 153 non-HCM age- and sex-matched controls	Single-centre	Yes (Siontis, 2024*)	ECG	ECG-based detection of HCM	AUC 0.96 (95% CI: 0.95–0.96); sensitivity of 87%; specificity of 91%	No	Retrospective
*Siontis, 2021^8^*	300 HCM pts ≤18 years vs. 18 439 age- and sex-matched non- HCM controls	Single-centre	No	ECG	ECG-based detection of HCM	AUC 0.98 (95% CI: 0.98–0.99); sensitivity of 92%; specificity of 95%	No	Retrospective
*Maanja, 2022^9^*	20 677 pts with at least one 12-lead ECG in January 2021 (derivation) and 15 147 pts with an ECG in January 2022 (testing)	Multicentre	No (internal testing)	ECG	Identify clinical markers distinguishing true and false positives to refine AI-ECG application for HCM	The clinical HCM-DETECT score had an AUC of 0.81 (95% CI: 0.73–0.87) for differentiating true- vs. false-positive results	No	Retrospective
*Siontis, 2024^10^	773 HCM pts vs. 3867 non-HCM controls	Multicentre	—	ECG	ECG-based detection of HCM	AUC 0.92 (95% CI: 0.91–0.93); diagnostic accuracy of 87%, sensitivity of 83%, and specificity of 88%	No	Retrospective
*Hillis, 2025^12^*	293 HCM vs. 2912 non-HCM controls	Multicentre	No	ECG	ECG-based detection of HCM	AUC, 0.97 (95% CI: 0.96–0.98); sensitivity of 68%; specificity of 99%	No	Retrospective
*Karra, 2024^13^*	12 281 pts (of which 1535 HCM pts)	Single centre	No	Echo	Identify HCM among individuals with LVH	AUC, 0.85; sensitivity 68%; specificity 99%	No	Retrospective
*Guo, 2022^14^*	123 HCM pts	Single-centre	No	CMR	Evaluate the performance of a DL-based method to automatically quantify LV function	EF from automatic segmentation identified HCM pts with sensitivity of 78% and specificity of 54%	No	Retrospective
*Chang, 2022^15^*	95 pts (of which 12 HCM)	Single centre	No	CMR	Test a DL algorithm for the automated measurement of native T1 and ECV	Agreement between DL and the reference: T1, *r* = 0.97 (95% CI 0.95–0.98); ECV, *r* = 0.99 (95% CI: 0.98–0.99)	No	Retrospective
*Augusto, 2021^16^*	60 HCM pts	Multicentre	No	CMR	Test an ML algorithm to automatically quantify MWT	Test–retest difference (mean ± SD): ML 0.7 ± 0.6 mm vs. experts 1.1 ± 0.9 mm to 3.7 ± 2.0 mm (*P* < 0.01)	No	Retrospective
*Fahmy, 2021^17^*	191 HCM pts	Multicentre	Yes	CMR	To accurately quantify LGE scar	%Scar_LGE-cine_ = 0.82 × %Scar_manual_, r = 0.84	No	Retrospective
*Baeßler, 2018^18^*	32 HCM pts and 32 controls	Single centre	No	CMR	To assess myocardial texture alterations in HCM on non-contrast T1-weighted images	Grey-level non-uniformity differentiated HCM pts from controls with sensitivity of 91%, specificity of 93%	No	Retrospective
*Zhang, 2021^19^*	1348 HCM pts	Multicentre (HCMR)	No (only independent testing)	CMR	To test a contrast agent–free technology to replace LGE	VNE showed strong agreement with LGE (ICC: 0.77–0.87; 95% CI, ≈20%)	Yes	Retrospective
*Yu, 2021^20^*	50 pts with HCM, 50 with HHD, 50 with UCM	Single centre	No	Echo	To differentiate multiple LVH aetiologies	HCM is significantly different in terms of brightness, SD, CoV, Skew, contrast7, and homogeneity5 (all *P* ≤ 0.005)	No	Retrospective
*Hwang, 2022^21^*	112 pts with HHD, 191 with HCM, 81 with AL-CA and 546 healthy subjects	Two centres	No	Echo	To differentiate multiple LVH aetiologies	AUC for HCM: 0.98 in test set, 0.99 in validation set	No	Retrospective
*Wu, 2022^22^*	74 AL-CA and 64 HCM pts	Single-centre	No	Echo	To differentiate CA from HCM	ML such as support vector machine (AUC 0.95, *P* = 0.477), random forest (AUC 0.97, *P* = 0.301), and gradient boosting machine (AUC, 0.98; *P* = 0.230) differentiated CA and HCM	No	Retrospective
*Duffy, 2022^23^*	23 745 pts	Multicentre	Yes	Echo	To quantify LVH and predict the aetiology of LVH	Accurate detection of CA (AUC, 0.79) and HCM (AUC, 0.89)	No	Retrospective
*Antonopoulos, 2021^24^*	149 pts 30 LVH, 61 HCM, 28 CA, and 30 healthy)	Single centre	No	CMR	Radiomics-based ML to classify LVH phenotypes	AUC 0.753 for multinomial classification of disease phenotype (normal vs. LVH vs. HCM vs. CA)	No	Retrospective
*Izquierdo, 2021^25^*	118 pts (35 LVNC, 25 HCM, 37 DCM, and 21 healthy)	Single centre	No	CMR	Radiomics-based ML to classify different cardiomyopathies	HCM-vs.-Rest AUC: 0.99	No	Retrospective
*Soto, 2022^26^*	2728 pts	Single centre	No	ECG Echo	To augment physician interpretation of LVH aetiology	High discriminatory ability in distinguishing HCM from hypertension with an AUC of 0.91, AUPRC of 0.78	Yes	Retrospective
*Wang, 2020^30^*	102 HCM pts with P/LP variants in *MYBPC3* and *MYH7*	Single-centre	No	CMR	To distinguish *MYBPC3* and *MYH7* HCM	Accuracy 92%; AUC 0.97 (95% CI: 0.96–0.97)	No	Prospective observational
*Morita, 2021^27^*	99 HCM pts	Single-centre	No	Echo	To predict genotype positivity	Mayo score + DCNN-derived probability vs. Mayo score alone (AUC, 0.86 vs. 0.72; *P* < 0.001). Toronto score + DCNN-derived probability vs. Toronto score alone (AUC 0.84 vs. 0.75; *P* = 0.03)	No	Retrospective
*Liang, 2021^28^*	102 HCM pts (training set) and 76 HCM pts (test set)	Two centres	Yes	Clinical Echo CMR Stress test	To predict genotype positivity	AUC of 0.92 (95% CI 0.85–0.99) in predicting genotype positivity in the test set NRI: *P* < 0.001 for the Toronto and Mayo score	Yes (upon request)	Retrospective
*Zhou, 2021^29^*	198 HCM pts	Single-centre	No	CMR	To predict genotype positivity	AUC, 0.80; sensitivity of 86%, specificity of 70%	No	Retrospective
*Liu, 2024^31^*	385 HCM tissue samples	Multicentre	No	Genetics	To identify HCM-related hub genes	—	No	Observational
*Lyon, 2019^34^*	85 HCM pts and 38 healthy volunteers	Single-centre	No	12-lead Holter ECG Echo CMR	To better stratify HCM pts risk	—	No	Retrospective
*Alis, 2020^41^*	64 HCM pts	Single-centre	No	CMR	To predict VTs	95% sensitivity; 93% specificity; 94% accuracy	No	Retrospective
*Kochav, 2021^40^*	183 HCM pts	Single-centre	No	Clinical Echo Stress test Genotype positivity	To predict MACE (death due to HF, heart transplant, and SCD)	ML model accuracy, 85%; sensitivity, 88%; specificity, 84%	No	Prospective
*Bhattacharya, 2021^35^*	831 HCM pts	Single-centre	No	Clinical Echo Stress test CMR	To distinguish AF and non-AF pts	Sensitivity, 74%; specificity, 70%; C-index of 0.80	No	Retrospective
*Smole, 2021^37^*	2302 HCM pts	Single-centre	No	Clinical Echo ECG Holter Genetics	To predict MACE (sustained VT, HF, ICD activation, SCD, cardiac death, and all-cause death)	The new ML model outperformed existing risk-stratification models for SCD, cardiac death, and all-cause death (higher AUC by 17%, 9%, and 1%, respectively)	Data may be available upon request	Retrospective
*Fahmy, 2021^39^*	2732 HCM pts	Single-centre	No	Clinical Echo	To predict HF progression at 5 years	AUC, 0.81 (95% CI, 0.76–0.86); accuracy, 74%; sensitivity, 80%; specificity, 72%	Yes^a^	Retrospective
*Pičulin, 2022^36^*	1860 HCM pts	Single-centre	No	Clinical ECG Echo CMR	To model the patient's clinical status up to 10 years ahead	The best-performing random forest model improved *R*^2^ from 0.3 to 0.6	No	Retrospective
*Rhee, 2024^33^*	2111 HCM pts	Two centres	Yes	Clinical Echo	To predict MACE (all-cause death, HF-adm, and stroke)	External validation cohort: AUROC for MACE of 0.77	No	Retrospective
*Al Wazzan, 2024^38^*	434 HCM pts	Two centres	No	LV longitudinal strain	To predict VAs	4 clusters identified	No	Retrospective
*Zhao, 2024^42^*	758 HCM pts (533 internal and 225 external cohort)	Multicentre	Yes	Clinical CMR	To predict MACE (VAs, SCD, HF, and AF-related stroke)	AUCs of 0.83 (internal) and 0.81 (external) The model outperformed HCM Risk-SCD model (AUC improvement of 23%)	No	Retrospective
*Abraham, 2022^44^*	EXPLORER-HCM data at week 30 (NCT03470545)	Multicentre	—	ECG	To characterize mavacamten effects beyond pVO2 increase and NYHA class reduction	The cluster analysis resulted in four main groups	No	Retrospective
*Siontis, 2023^43^*	13 HCM pts (216 ECGs) from PIONEER-OLE 2600 age- and sex-matched controls	Multicentre	—	ECG	To evaluate response to mavacamten	Mean HCM score decreases during mavacamten treatment: 0.80–0.45 for Mayo and 0.70–0.35 for USCF algorithms	No	Longitudinal
*Suppah, 2025^45^*	ECG: 27 HCM pts Echo: 58 HCM pts	Single-centre	No	ECG Echo	To evaluate response to mavacamten	Notable reductions in HCM phenotype severity (median, 29.7% to 0.4%, *P* = 0.001) and diastolic dysfunction (median, 2 to 0, *P* = 0.004)	No	Longitudinal

AF, atrial fibrillation; AL-CA, light-chain cardiac amyloidosis; AUC, area under the curve; AUPRC, area under the precision-recall curve; CA, cardiac amyloidosis; CI, confidence interval; CMR, cardiac magnetic resonance; CoV, coefficient of variation; DCNN, deep convolutional neural network; DL, deep learning; DCM, dilated cardiomyopathy; Echo, echocardiography; ECG, electrocardiogram; EF, ejection fraction; ECV, extracellular volume; HF, heart failure; HHD, hypertensive heart disease; HCM, hypertrophic cardiomyopathy; ICC, intraclass correlation coefficient; ICD, implantable cardioverter-defibrillator; LGE, late gadolinium enhancement; LV, left ventricle; LVH, left ventricular hypertrophy; LVNC, left ventricular non-compaction cardiomyopathy; LVOT, left ventricular outflow tract; MACE, major adverse cardiovascular events; ML, machine learning; MWT, maximum wall thickness; MYBPC3, myosin-binding protein C; MYH7, β-myosin heavy chain; NYHA, New York Heart Association; oHCM, obstructive hypertrophic cardiomyopathy; P/LP, pathogenic or likely pathogenic; PPG, photoplethysmography; pts, patients; pVO2, peak oxygen consumption; RF, random forest; SCD, sudden cardiac death; SD, standard deviation; UCM, uraemic cardiomyopathy; UCSF, University of California, San Francisco; VAs, ventricular arrhythmias; VNE, virtual native enhancement; VTs, ventricular tachyarrhythmias; VUS, variant of uncertain significance.

^a^ https://dataverse.harvard.edu/dataset.xhtml?persistentId=doi:10.7910/DVN/FFNLPE.

## Applications of artificial intelligence in hypertrophic cardiomyopathy

### Early detection of HCM

AI algorithms analyse cardiac signals by learning abstract data representations associated with different features of disease, such as on electric vectors, wall thickness, distribution of hypertrophy, myocardial texture, and ventricular morphology.^4^ The ECG is a non-invasive, primary, inexpensive tool to diagnose cardiac abnormalities, and it is therefore used as an early screening tool for cardiovascular diseases.

In 1998, Ouyang *et al*. conducted one of the earliest AI-ECG studies on 79 patients with HCM, using supervised ML for diagnosing the hypertrophic portions of HCM.^5^ In 2019, Tison *et al*. developed an AI model combining ML and DL to analyse 36 186 raw ECG recordings, aiming to estimate cardiac structure parameters like LVH and detect different diseases. Among these, HCM was identified with good discrimination [area under the curve (AUC) 0.91], mainly driven by ST-T alterations in lead V1, prolonged P wave, QT and PR intervals, and QRS features in lead aVR.^6^

In 2020, the Mayo Clinic developed an AI-ECG model for HCM detection from standard 12-lead ECG alone.^7^ Raw ECG data from 2448 HCM patients and 51 153 non-HCM age- and sex-matched controls were used to test and train a CNN. ECGs with the presence of ventricular pacing or left bundle branch block were not included. The AUC in the testing dataset was 0.96 [95% confidence interval (CI) 0.95–0.96], performing particularly well in younger patients (sensitivity, 95%; specificity, 92%). This model was later tested as a screening tool in a paediatric HCM population, demonstrating accurate detection of HCM regardless of sex or genotype.^8^ However, a key limitation in early detection algorithms is the high false-positive rates and, notably, the challenge of distinguishing true early HCM from other causes of LVH, such as aortic stenosis or hypertensive heart disease. Ko *et al*.^7^ specifically trained their CNN model to differentiate HCM from aortic stenosis and LVH, partially addressing this critical diagnostic step. This distinction is essential for clinical accuracy, particularly when applying AI to general population screening in terms of healthcare costs. Given the potential high false-positive rates of AI-ECG diagnosis of HCM, Maanja *et al*. developed a clinical score (HCM-DETECT) incorporating variables such as age, coronary artery disease, and prior pacemaker or valve surgery to refine prediction.^9^ Their findings highlight that many false-positive results are driven by non-HCM-related LVH, QRS widening, or ST-segment deviations—features that reduce specificity and risk over-diagnosis. The tandem use of AI with clinical filtering helps mitigate this issue, but the risk of overburdening healthcare systems with unnecessary follow-up remains a concern.

Additionally, these studies have several limitations that restrict their immediate generalizability. First, these AI-ECG models have been tested in single-centre, retrospective cohorts, raising concerns about their overfitting. To address this, Siontis *et al*. recently conducted an external validation study applying the Mayo Clinic AI-ECG algorithm to three independent international cohorts.^10^ This study included 773 patients with HCM and 3867 controls across diverse ethnic backgrounds (54.6% East Asian, 43.2% White, 2.2% Black) and demonstrated that the algorithm maintained high performance (AUC, 0.92; sensitivity, 83%; specificity, 88%), providing reassuring evidence of external validity. Nevertheless, prospective clinical studies are still needed to confirm its utility in real-world screening and to evaluate cost-effectiveness and clinical impact.

Wearable devices are non-invasive technologies designed to monitor physiological parameters continuously or intermittently. These include commercially available devices such as smartwatches with ECG functionality (e.g. Apple Watch®), adhesive ECG patches (e.g. Zio® ECG monitors), chest strap monitors, and wrist-worn fitness bands. Such devices are increasingly used in clinical practice for remote monitoring of cardiac rhythm. Research is currently exploring the use of wearable biosensors for structural heart disease screening. For example, Green *et al*. developed an ML model using photoplethysmography recordings from a smartwatch-like device to detect obstructive HCM in a cohort of 19 patients and 64 healthy controls.^11^ These findings are still at the research stage and require further validation in larger, real-world populations before being adopted as clinical screening tools.^12^

While transthoracic echocardiography (TTE) is a cornerstone for the initial evaluation of patients with LVH, the application of AI to echocardiographic imaging for HCM detection remains extremely limited. To date, only one study has explored this approach by training a CNN on standard TTE views (parasternal long axis and apical four-chamber) from over 12 000 patients.^13^ The model achieved an AUC of 0.85, with high specificity (99%) but moderate sensitivity (68%) for identifying HCM among patients with septal hypertrophy.

AI-enhanced CMR provides valuable insights into HCM diagnosis by enabling more precise and automated image interpretation. There are already commercially available DL algorithms that allow automated assessment of left ventricle (LV) volume and function^14^ as well as T1 or extracellular volume measurement in HCM patients.^15^ In a preliminary study, ML-based maximum wall thickness (MWT) measurement in HCM was superior to human experts; interestingly, this has direct implications, as it has been shown that a clinical trial using ML would need 2.3 times (1.6–4.6) fewer patients to detect a 2 mm MWT change.^16^

Myocardial scar quantified by late gadolinium enhancement (LGE) has a diagnostic and prognostic role in HCM; however, manual segmentation of myocardial borders and scarred regions is highly prone to reader variability. Automated image segmentation using deep CNNs can offer standardized LGE analysis and reduce the time and effort required for manual contouring.^17^ One of the most promising applications of AI is the possibility of contrast-sparing HCM diagnosis. This may lead to the development of a contrast–free technology to replace LGE for faster and cheaper CMR scans.^18^ ML models can indeed be used as a scar screening tool prior to gadolinium administration. Virtual native enhancement, a new DL–driven CMR technology, can generate images that closely resemble conventional LGE without the need for gadolinium-based contrast agents, achieving high agreement with LGE itself.^19^

### Differential diagnosis

Using pattern recognition, AI can distinguish HCM from other forms of LVH.^20^ A DL algorithm has been developed to help differentiate common causes of LVH, including hypertensive heart disease (HHD), HCM, and light-chain cardiac amyloidosis (CA), using standard TTE images.^21^ Similarly, ML combined with 2D-speckle tracking echocardiography has performed well by integrating plentiful variables to identify the most discriminative predictors.^22^ A study by Duffy *et al*. assessed the accuracy of a DL algorithm in quantifying LVH and differentiating its aetiology between CA and HCM.^23^ The algorithm was trained and tested on video echocardiograms. In 23 745 patients, the DL model accurately measured LV dimensions and classified CA and HCM. The algorithm also demonstrated high accuracy in external validation sets, distinguishing HCM from other causes of LVH with an AUC of 0.98.^23^ However, these studies are constrained by limited sample sizes, restricted clinical settings, and a lack of publicly available datasets; most rely on retrospective analyses without prospective external validation, making their translation to routine clinical practice still premature.

CMR is often superior to echocardiography in identifying areas of segmental hypertrophy, not reliably visualized by echocardiography. Myocardial radiomic phenotyping has proved successful in distinguishing healthy from hypertrophic myocardium and can also differentiate LVH aetiology, including HCM or CA.^24,25^

Unlike previous studies, Soto and colleagues proposed the first DL model that integrates both ECG and echocardiogram data to differentiate HCM from HHD.^26^ By comparing various fusion strategies for combining multimodal data with single-modality models, the authors demonstrated that integrating spatio-temporal information from both modalities significantly improves classification performance, achieving an AUC of 0.91.^26^ Although the study lacked external validation on independent cohorts, the authors addressed this limitation by open-sourcing their code and releasing the trained models, promoting reproducibility and supporting further research.

### Deeper genetic insights

Genetic testing is essential for guiding family screening strategies and has significant prognostic and diagnostic value in HCM. However, traditional genetic testing is time- and resource-consuming, limiting its widespread application. In this context, AI can dramatically speed up genome sequencing, enhancing accuracy and reducing errors in identifying genetic variants. Few studies have explored DL/ML algorithms for mutation-risk prediction and genotype positivity in HCM.^27–29^ In a cohort of 99 HCM patients, deep CNN analysis of TTE images outperformed the Mayo and the Toronto genotype score predictors, and combining both models further improved predictive accuracy.^27^ Similarly, an ML model constructed with clinical and cardiac imaging data was trained to predict genotype positivity in a cohort of 102 (training set) and 76 (test set) HCM patients.^28^ The ML model demonstrated an AUC of 0.92 (95% CI, 0.85–0.99) in predicting genotype positivity in the test set, significantly outperforming the Toronto and the Mayo scores.^28^ Radiomic CMR analysis may play a role not only in predicting genotype positivity,^29^ but also in effectively distinguishing between different genetic subtypes of HCM, such as β-myosin heavy chain (*MYH7*) and β-myosin-binding protein C (*MYBPC3*).^30^

Finally, a recent study applied an ML-driven multi-cohort analysis to identify a diagnostic gene signature for HCM, focusing on early-stage disease.^31^ The Authors identified 27 hub genes and developed a stable diagnostic model. Moreover, the study revealed immune-related mechanisms, including differential immune-cell infiltration patterns between high- and low-risk groups, highlighting novel potential therapeutic targets.^31^

Several limitations should be acknowledged, including the lack of external validation in most cases, potential selection bias towards patients already undergoing genetic testing, and limited diversity in terms of race and clinical presentation. Additionally, genotype classifications are based on current knowledge and may evolve over time, raising questions about long-term model reliability and generalizability.

In parallel, AI-based bioinformatics approaches have emerged to address the growing number of variants of uncertain significance identified through large-scale sequencing. For example, Burghardt and Ajtai developed a neural/Bayes framework that models the structure-function relationships of missense mutations in key sarcomeric proteins, such as MYH7 and MYBPC3.^32^ Such methods hold promise for improving genetic interpretation, accelerating risk stratification, and informing early clinical decision-making as genomic data continue to expand.

### Risk stratification

Most patients with HCM remain asymptomatic throughout their lives, and only a small proportion of patients develop serious adverse outcomes such as end-stage heart failure (HF), cardiovascular death, and SCD. Current risk-stratification strategies are based on a limited number of elements and offer incomplete prediction (focusing mainly on arrhythmic risk). In this context, by thoroughly incorporating multidimensional data and factors that interact in linear and nonlinear manners, the ML-based methodology may provide a model with significantly enhanced prediction performance.^33^

In a study by Lyon *et al*., by applying ML on 12-lead Holter ECG, the Authors were able to classify HCM patients into four distinct phenotypes of ventricular remodelling, each associated with different levels of arrhythmic risk.^34^ Importantly, the study identified two potential mechanisms—one related to conduction abnormalities and the other to ion channel remodelling—that could explain HCM heterogenic manifestations.^34^ Similarly, other ML-based models have proved to be effective in identifying atrial fibrillation (AF)^35^ and in predicting HCM disease progression in terms of adverse remodelling,^36^ risk of ventricular arrhythmias (VAs),^37,38^ and progression to HF.^37,39^ In a prospective study by Kochav *et al*., ML models were applied to a cohort of 183 patients with HCM.^40^ During a median follow-up of 2.2 years, 33 subjects (18%) developed the primary outcome (a composite of heart transplantation, death due to HF, and SCD), the majority of whom (*n* = 28) underwent heart transplantation. The authors determined 20 predictive features (clinical, imaging, and genetics) based on random forest classification and a priori knowledge and developed 4 ML models, all significantly outperforming the reference model in predicting outcomes.^40^

Pičulin *et al*. were the first to develop an ML-based model aimed at predicting long-term disease progression.^36^ Their approach integrates six independent regression models to forecast key clinical parameters, including ventricular function and chamber dimensions, up to ten years ahead. Notably, their models demonstrated superior predictive performance compared to expert estimations in five out of six targets, highlighting the potential of AI to support longitudinal management in HCM.

CMR is useful in identifying high-risk HCM subgroups with extensive fibrosis, thin-walled scarred LV apical aneurysms, and end-stage systolic dysfunction. Radiomic characterization of CMR data offers deeper insights into myocardial tissue properties, thereby enhancing risk stratification. ML-based texture analysis of LGE has shown strong performance in distinguishing HCM patients with and without VTs.^41^ In a recent multicentre study involving 758 patients with HCM, Zhao *et al*. developed an ML-based framework integrating LGE and CMR myocardial strain with clinical parameters to predict major adverse cardiovascular events, including VAs, HF, AF-related stroke, and SCD.^42^ The model achieved robust performance with AUCs of 0.83 in internal validation and 0.81 in an external multicentre cohort, significantly outperforming the traditional HCM Risk-SCD model AUC by 23%. Importantly, the study highlighted nonlinear associations between the extent of LGE, impaired myocardial strain, and elevated event risk.^42^

However, many of these models were developed using retrospective, single-centre, or demographically homogeneous cohorts. Small event numbers, short follow-up durations, and limited external validation further reduce clinical applicability. Reproducibility remains a major concern, as most models do not share their code or use publicly available datasets. Finally, the “black box” nature of many AI models, along with reliance on data preprocessing or synthetic augmentation, raises questions about interpretability and real-world implementation. Prospective, multicentre validation and standardized methodologies are essential next steps to ensure clinical translation.

### Evaluation of treatment efficacy

Recent advances in the treatment of HCM have significantly broadened the therapeutic options available, particularly with the introduction of cardiac myosin inhibitors (CMIs), mavacamten, and aficamten. These novel agents directly address myocardial hypercontractility, a hallmark of the disease, delivering both clinical and symptomatic benefits. As these therapies gain traction, AI emerges as a key tool for patient selection and treatment response evaluation.

In a study by Siontis *et al*., two AI-ECG algorithms (University of California-San Francisco [UCSF] and Mayo Clinic) were developed and trained independently at two institutions to format 216 serial ECG data from the PIONEER-OLE trial (i.e. the open-label extension of the mavacamten phase 2 trial).^43^ In the validation cohorts, both algorithms exhibited similar performance for HCM diagnosis, and exhibited mean HCM score decreases during mavacamten treatment: 0.80–0.45 for Mayo and 0.70–0.35 for USCF algorithms.^43^ Interestingly, AI-ECG HCM scores correlated with disease status, haemodynamic changes in left ventricular outflow tract (LVOT) gradients, and serum N-terminal pro–B-type natriuretic peptide levels in patients with obstructive HCM on mavacamten treatment, even when ECG changes were less apparent.^43^

An additional post-hoc ML analysis of the EXPLORER-HCM trial grouped patients according to their improvement status during mavacamten treatment, using unsupervised hierarchical clustering. The cluster analysis revealed four distinct patient groups based on their responses to treatment.^44^ Two clusters (Groups 1 and 2), which included the majority of mavacamten-treated patients (88% and 85%, respectively), showed improvements in several clinical endpoints: Group 1 met both primary and secondary endpoints, while Group 2 showed meaningful benefits in secondary outcomes despite not meeting the primary endpoint. In contrast, Groups 3 and 4 were predominantly placebo-treated patients (95% and 90%, respectively), with Group 3 meeting only the primary endpoint and Group 4 showing no significant improvement across measures. These findings highlight that mavacamten may provide broader clinical benefits than captured by the primary endpoint alone, reinforcing the value of multidimensional treatment assessment in HCM.^44^

In addition to post-hoc analyses of randomized trials, real-world studies have further supported the value of AI tools in treatment monitoring. In a recent single-centre analysis, AI-ECG and TTE parameters were evaluated in obstructive HCM patients treated with mavacamten for at least six months.^45^ AI-ECG detected a reduction in HCM phenotype severity (from 29.7% to 0.4%, *P* = 0.001) and diastolic dysfunction grade (from 2 to 0, *P* = 0.004), while TTE showed parallel improvements in LVOT gradients (from 71 to 0 mmHg, *P* = 0.001), left atrial volume index, and diastolic function parameters (*P* = 0.001 for both).^45^ Interestingly, these benefits appeared independent of LVOT gradient reduction, suggesting that mavacamten's disease-modifying effects extend beyond simple haemodynamic unloading.

In summary, AI-enhanced ECG analysis has demonstrated potential in assessing treatment response to myosin inhibitors like mavacamten, establishing correlations with disease progression, biomarker profiles, and clinical outcomes in HCM.

### AI-driven *in silico* trials and digital twin solutions

While AI applications have shown encouraging results in HCM, these tools often operate on isolated datasets and lack integration across different biological levels. This limits their ability to fully capture the complexity of HCM, which spans diverse genetic backgrounds, phenotypes, and clinical trajectories.

To overcome these limitations, *in silico* trials and digital twin technologies have emerged as promising innovations. *In silico* trials are computer-based simulations that model disease progression or treatment outcomes using patient-specific data. They offer a cost-effective and ethical alternative to certain stages of clinical research, enabling virtual testing of therapies before their application in patients. Digital twins expand on this concept by creating a comprehensive, virtual tool that integrates coherently and dynamically the clinical data acquired over time for an individual using mechanistic and statistical models.^46^ In cardiovascular medicine, digital twin platforms have been explored for applications such as arrhythmia risk prediction, procedural planning for atrial and VA ablation, simulation of HF progression, and non-invasive assessment of cardiovascular dynamics through wearable sensors.^46^ These technologies offer significant advantages, including the ability to test interventions *in silico* before applying them to real patients, optimize treatment strategies, and reduce costs. However, they also face limitations, such as the need for large, high-quality datasets, computational complexity, regulatory challenges, and the risk of over-reliance on models that may not fully capture biological variability.

**Figure 3:**
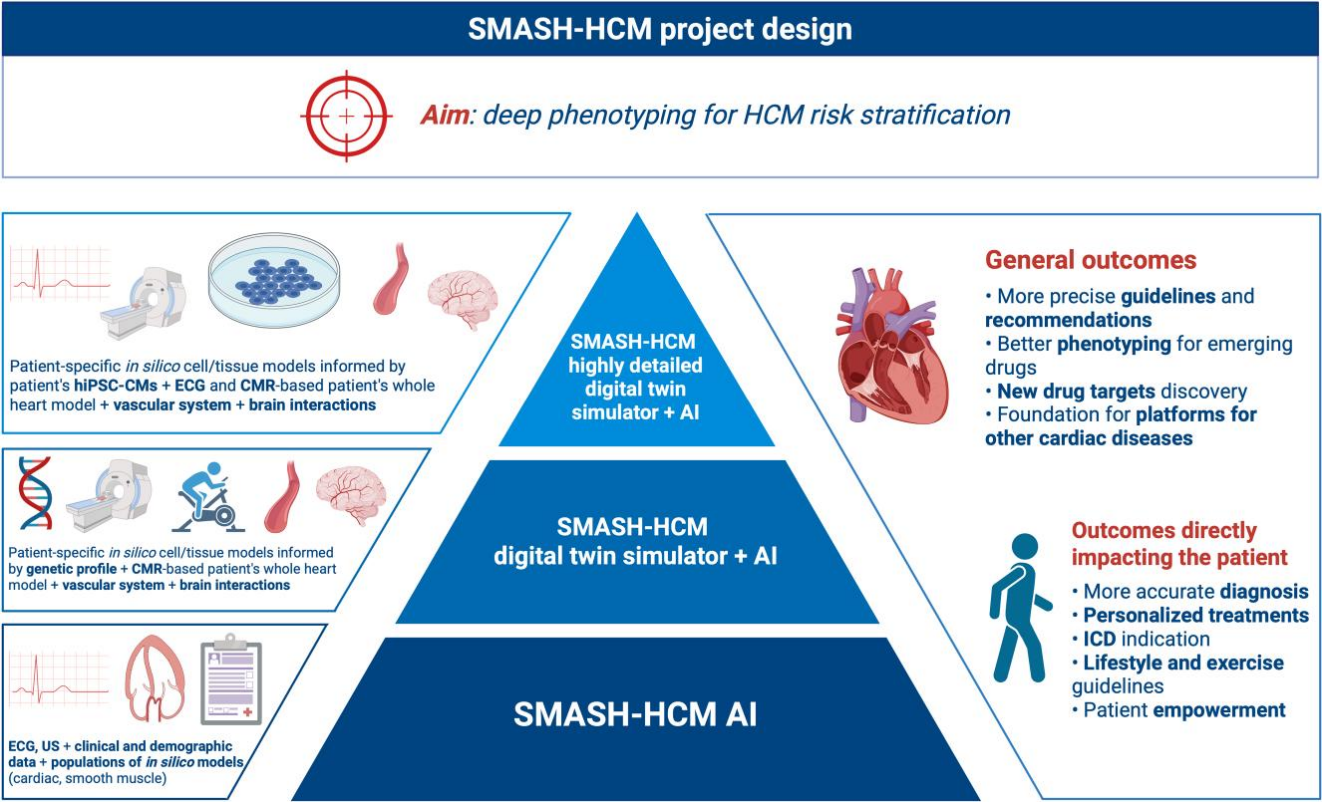
SMASH-HCM project design. The SMASH-HCM project introduces a three-level digital twin platform designed to maximize risk stratification. At the first level, it integrates standard clinical data like ECGs and echocardiography with AI-driven insights from combined clinical, *in vitro*, and synthetic population data. The second level incorporates extended patient data, enabling the development of biophysical digital twin models. At the third level, the platform uses comprehensive phenotyping to refine the digital twin, providing advanced tools for personalized treatment and improved management. ECG, electrocardiogram; AI, artificial intelligence; LVH, left ventricular hypertrophy; CMR, cardiac magnetic resonance; HCM, hypertrophic cardiomyopathy; hiPSC-CM, human-induced pluripotent stem cell-derived cardiomyocytes; ICD, implantable cardioverter defibrillator; US, ultrasound.

An important example of this approach in HCM is the SMASH-HCM project (*Stratification, Management, and Guidance of Hypertrophic Cardiomyopathy Patients using Hybrid Digital Twin Solutions*) (https://smash-hcm.eu). SMASH-HCM aims to build a digital twin platform that combines AI models with mechanistic simulations to improve risk stratification, treatment personalization, and patient self-management. The project is structured into three levels of deep phenotyping (*Figure 3*). At the first level, AI/ML models will be developed to analyse clinical data, including ECGs and imaging data, to provide initial stratification and lifestyle recommendations. Secondly, AI/ML models will integrate advanced diagnostics such as CMR, genetic profiling, and patient-specific whole-heart and systemic models to refine risk stratification and personalize treatment recommendations. The third level will incorporate human-induced pluripotent stem cell-derived cardiomyocytes or other patient-specific *in vitro* data, along with computational models of cells and *in vitro* mimicking tissues, enabling detailed mechanistic insights into cardiac function, disease progression, and treatment response.

Importantly, SMASH-HCM goes beyond technical development by embedding patient engagement and self-management tools into its design, including health literacy strategies, behaviour change frameworks, and gamification elements to enhance patient adherence and empowerment. While still under development, the PILOT clinical trial will be crucial in validating SMASH-HCM's real-world impact, testing its feasibility across diverse patient profiles and clinical settings. If successful, SMASH-HCM could set a new standard for integrated, AI-driven care in HCM, potentially extending this model to other cardiovascular diseases.

## Clinical implementation and regulatory hurdles

Despite the increasing momentum around AI applications in HCM, real-world clinical implementation remains limited. A major barrier is the lack of regulatory approval for many AI-based tools, particularly those tailored to HCM. As of May 2025, the U.S. Food and Drug Administration (FDA) has authorized over 1000 AI-enabled medical devices, the majority of which are focused on radiology and are not HCM-specific (e.g. us2.v1). To date, Viz HCM is the only FDA-cleared algorithm specifically approved for the detection of HCM. It analyses 12-lead ECGs in real time to flag suspected HCM cases and facilitates timely referral to specialist care.

To support broader clinical adoption, AI systems must comply with evolving regulatory and ethical standards designed to ensure safety, transparency, and fairness in healthcare. In the United States, the FDA has shown growing engagement with AI across domains such as digital health and drug development. In 2025, the FDA's Center for Drug Evaluation and Research released draft guidance titled ‘Considerations for the Use of Artificial Intelligence to Support Regulatory Decision Making for Drug and Biological Products.’ This guidance outlines a risk-based, trustworthy, and transparent framework for integrating AI into the drug life cycle and regulatory submissions.

Meanwhile, in the European Union (EU), the regulatory landscape has been reshaped by the EU Artificial Intelligence Act (https://artificialintelligenceact.eu), which introduces a tiered, risk-based classification for AI systems, including those in healthcare. High-risk AI tools—such as those involved in diagnosis, treatment planning, or prognosis—will be required to meet strict criteria around transparency, human oversight, robustness, and accountability. Complementing this, the Ethics Guidelines for Trustworthy AI published in 2019 by the EU High-Level Expert Group on AI articulate seven core principles for ethical AI: human agency and oversight, technical robustness and safety, privacy and data governance, transparency, non-discrimination and fairness, societal and environmental well-being, and accountability. These guidelines serve as a blueprint for the responsible design, deployment, and auditing of AI systems in clinical practice.

## Drawbacks of AI models

While the majority of published AI studies report promising results, it is important to recognize that several limitations continue to hinder their clinical translation. Moreover, negative or failed AI models are rarely published, potentially introducing a publication bias. A primary challenge is the need for large, high-quality datasets to effectively train AI models, which can be particularly difficult to obtain in HCM. To address this, collaborative efforts among research institutions, hospitals, and patient registries can facilitate data pooling. Even when datasets are available, another critical limitation is the lack of model transparency and reproducibility. Many published studies do not disclose their trained models, making it impossible to replicate or further exploit their results. As a result, researchers must retrain models from scratch, which requires access to the original data. For example, to address this, SMASH-HCM will generate a library of models, making them fully disclosed whenever possible, and will ensure that all shared data and tools comply with the FAIR principles (Findable, Accessible, Interoperable, and Reusable), promoting transparency, interoperability, and long-term usability across the research community. Additionally, it will develop a structured and accessible “surfable” encyclopaedia of current AI models, serving as a foundational knowledge base for researchers in the field.

An additional concern is data leakage,^47^ where unintended information sharing between training and test sets, the use of unavailable predictors at inference time, or the inclusion of future data in training artificially inflates model performance. Biases—including selection bias, cherry-picking of results, overtraining, HARKing (hypothesizing after results are known), and confirmation bias—are common pitfalls in AI research. Ensuring transparent reporting, pre-registered study protocols, and independent validation on external datasets can help mitigate these issues. Overfitting remains another major limitation, where AI models perform well on training data but struggle to generalize to new, unseen cases. Implementing robust validation techniques, such as cross-validation or validation in external cohorts, can help mitigate this issue.

A practical drawback, particularly relevant for screening applications such as AI-ECG, is the high rate of false positives when applied in real-world or low-prevalence populations.^9^ This can lead to over-referral, increased healthcare workload, and unnecessary diagnostic procedures. AI models should be accompanied by appropriate clinical filtering tools or context-specific thresholds to reduce this unintended burden.

Finally, the real-world application of AI raises ethical questions, questions about trustworthiness (including fairness, bias, and robustness), and privacy concerns, necessitating the development of regulatory frameworks and guidelines to govern its use.^48,49^

## Future developments

Looking ahead, the integration of AI holds great potential to address many of the current limitations in HCM management.^50^ Most AI models are still built on retrospective, single-centre data. This limits their generalizability, especially in underrepresented patient populations such as younger individuals, ethnic minorities, and those with atypical or non-sarcomeric forms of HCM. As shown in recent work,^10^ subgroup-specific performances should be explicitly evaluated to ensure fairness and avoid perpetuating healthcare disparities. Future efforts must focus on training and validating AI models in larger, multicentre, and globally diverse cohorts, with subgroup-specific performance reporting.

Importantly, the current dynamic landscape of HCM—particularly with the advent of CMIs—poses both challenges and opportunities for risk stratification. Although CMIs have demonstrated benefits in obstructive HCM, management of the non-obstructive form remains difficult. AI models could play a transformative role by accelerating the identification of responder subgroups, optimizing inclusion criteria, and informing the design of adaptive clinical trials. Furthermore, models trained on large, longitudinal cohorts may help prioritize surrogate endpoints for regulatory validation, improving the speed and precision of therapeutic development. AI could also improve our ability to identify early biomarkers, clinical features, or genetic profiles associated with progression to severe disease. For example, distinguishing between asymptomatic gene mutation carriers who may never develop clinical HCM and those at higher risk for symptomatic progression remains a major unmet need. Currently, all mutation carriers are monitored throughout life, regardless of risk. AI-based tools could enable personalized follow-up strategies by stratifying carriers based on their predicted lifetime risk.

Beyond genomics, several other domains are rapidly evolving. AI-based imaging tools are being developed to detect subtle phenotypic markers, automate quantification, and track disease progression over time. ML approaches to genotype-phenotype correlations are beginning to uncover complex, nonlinear relationships that could inform prognosis, family screening, and personalized therapy. Meanwhile, wearable technologies and remote monitoring systems powered by AI offer a promising avenue for continuous, real-time data collection, enabling earlier identification of arrhythmic risk or decompensation in outpatient settings. At the frontier of these developments are digital twin technologies and *in silico* trials, which represent a novel paradigm for precision medicine. Although clinical validation is still ongoing, SMASH-HCM exemplifies the potential of hybrid models that merge data-driven and mechanistic approaches to develop a digital twin platform for HCM.

Crucially, real-world implementation of AI tools depends not only on technical performance but also on clinical trust, usability, and integration into existing workflows. Many healthcare professionals remain hesitant to adopt AI unless its outputs are interpretable and aligned with clinical needs. Therefore, clinicians should be actively involved in designing user interfaces, and close, iterative collaboration between AI developers and frontline physicians is essential to ensure adoption.

## Conclusions and learning points

The integration of AI into HCM management may represent a significant advancement in precision and personalized medicine, enhancing diagnostic accuracy, risk stratification, and treatment efficacy. AI-driven *in silico* trials, such as the SMASH-HCM digital twin platform, exemplify the potential of AI to integrate multiscale data, and biophysical simulations, for deep phenotyping, personalized risk assessment, and developing patient-specific treatment strategies. Advancing AI for HCM will require a shift from retrospective, one-size-fits-all models to dynamic, explainable, and equitable solutions that leverage both real-world data and computational simulation. These advancements, combined with an increasing number of disease-modifying therapies, may revolutionize the landscape of HCM management.

## Data Availability

No new data were generated or analysed in support of this research.
